# Combined effects of PLK1 and RAS in hepatocellular carcinoma reveal rigosertib as promising novel therapeutic “dual-hit” option

**DOI:** 10.18632/oncotarget.23188

**Published:** 2017-12-11

**Authors:** Peter Dietrich, Kim Freese, Abdo Mahli, Wolfgang Erwin Thasler, Claus Hellerbrand, Anja Katrin Bosserhoff

**Affiliations:** ^1^ Institute of Biochemistry, Emil-Fischer-Zentrum, Friedrich-Alexander-University Erlangen-Nürnberg, Erlangen, Germany; ^2^ Biobank o.b. HTCR, Department of General Visceral- and Transplantation Surgery, Ludwig-Maximilians-University München, München, Germany; ^3^ Comprehensive Cancer Center (CCC) Erlangen-EMN, Erlangen, Germany

**Keywords:** HCC, HRAS, PLK1, RAS, rigosertib

## Abstract

Inhibition of RAS-RAF-ERK-signaling is a major mechanism mediated by the multi-kinase inhibitors sorafenib and regorafenib, the only effective therapeutic approaches for advanced hepatocellular carcinoma (HCC). This underlines the importance of RAS-RAF-ERK-signaling in HCC. Most RAS isoforms were not yet described to play crucial roles in HCC. However, several studies indicate that the HRAS isoform can function as potent oncogene in HCC, but pharmacologic RAS inhibition has not yet been investigated. Moreover, the cell cycle promoting polo-like kinase 1 (PLK1) is an increasingly recognized therapeutic target in HCC that can be activated by RAS-RAF-signaling.

A recently developed small molecule inhibitor, ON-01910 ("rigosertib", RGS), was shown to interfere with both RAS- and PLK1-signaling. The aim of this study was to analyze the effects of RGS in HCC and to assess PLK1 and HRAS expression in HCC. RGS treatment reduced cell proliferation and induced cell cycle arrest in human HCC cell lines *in vitro*. Moreover, RGS strongly inhibited both ERK- and AKT-activation in HCC cells, indicating disruption of RAS-signaling.

Analysis of HCC patient data showed that PLK1 and HRAS expression levels are upregulated during HCC development and in advanced HCC, respectively. High expression levels of PLK1 significantly correlated with poor patient survival. Moreover, high expression of both PLK1 and HRAS revealed combined effects on patient outcome. This underscores the importance of these genes and associated pathways in HCC. We newly demonstrate the therapeutic potential of RGS in HCC by inhibition of both PLK1 activation and major RAS-pathways, revealing a novel therapeutic “dual-hit” approach for HCC.

## INTRODUCTION

Hepatocellular carcinoma (HCC) is one of the leading causes of cancer-related mortality worldwide [1, 2]. By now, sorafenib is the only successful first-line therapeutic option for patients with advanced disease [3–5]. The RAS-RAF-ERK-pathway serves as the major target of the effects mediated by the multi-kinase inhibitors sorafenib and regorafenib, underlining the importance of MAPK-signaling in HCC [3, 6, 7]. However, disease progression after sorafenib treatment occurs in the majority of treated patients [5, 8, 9]. Regorafenib is the only approved systemic second-line treatment shown to provide a modest survival benefit (2.8 months) in HCC patients progressing on sorafenib treatment [10]. Therefore, novel and more successful therapeutic approaches are urgently needed [2, 3].

The MAPK-signaling-associated polo-like kinase 1 (PLK1), a protein involved in promoting cell cycle progression, is increasingly recognized as attractive therapeutic target in HCC [11–17]. ON-01910 (“rigosertib”, “RGS”), a novel benzyl styryl sulfone, has been considered to inhibit PLK1 as major mechanism of action [18]. However, most recently, Athuluri-Divakar *et al.* found that RGS actually acts as a RAS-mimetic that binds to the RAS binding domains (RBDs) of RAS effectors. RGS was shown to reduce the transforming powers of RAS and inhibited RAS-signaling [18]. While the RAS isoforms NRAS and KRAS are uncommonly mutated and therefore not much recognized as oncogenic targets in HCC [19], HRAS alterations were found in murine hepatoblastomas and adjacent HCC [20]. Moreover, activating HRAS mutations were recently detected in HCC developed in mice with non-alcoholic fatty liver disease [21], which is increasingly recognized as promotor of hepatocarcinogenesis [1].

The aim of this study was to assess the combined expression and function of PLK1 and HRAS in HCC. Moreover, we analyzed the effects of RGS on human HCC cells and demonstrated that this small molecule strongly reduced cell proliferation by affecting cell cycle progression and inhibition of major RAS-effector pathways.

## RESULTS

### Effect of rigosertib on viability of human HCC cells

**Figure 1:**
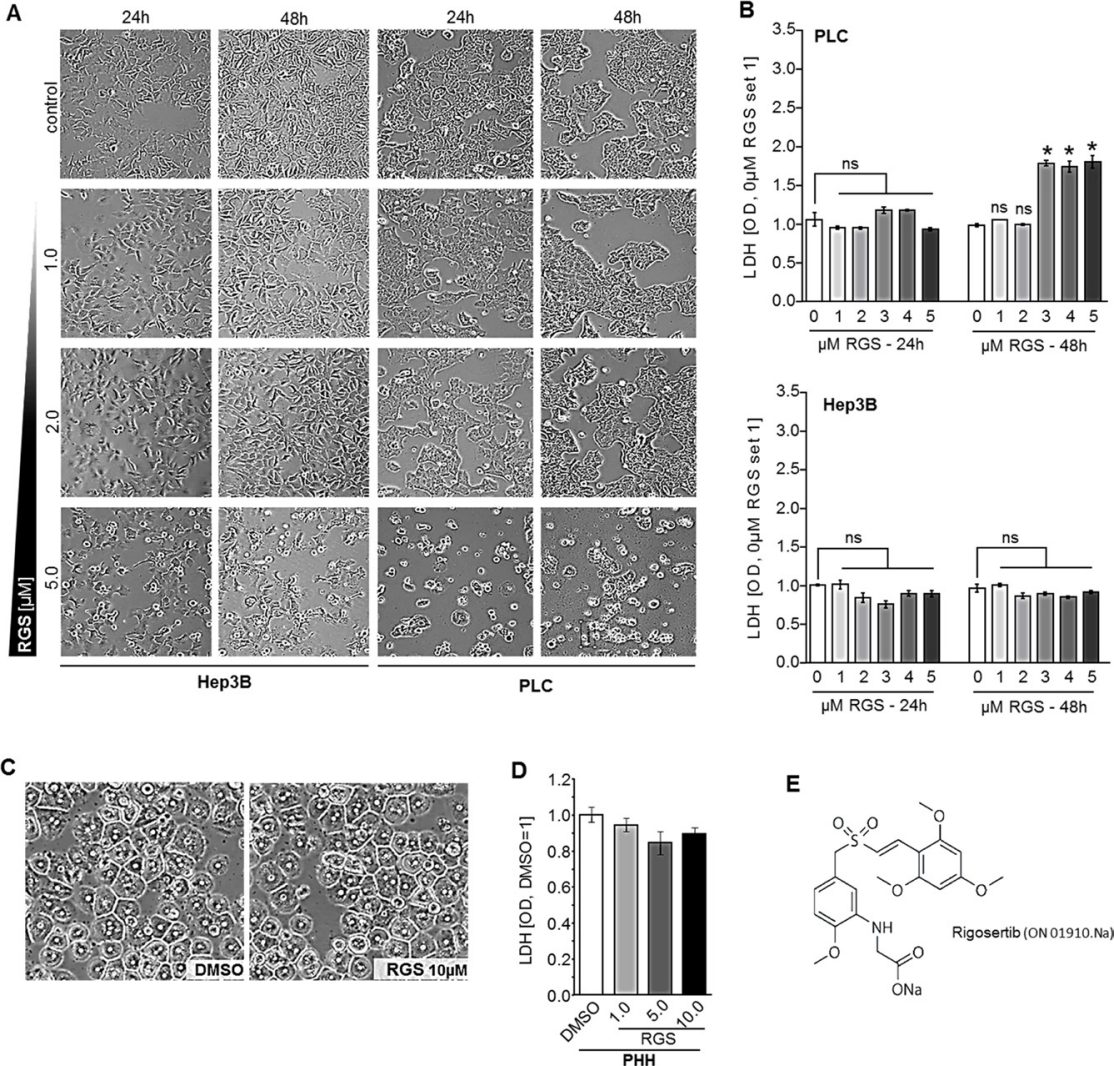
Effect of rigosertib on viability of human HCC cells (**A**) PLC and Hep3B cells were treated with DMSO as compared to rigosertib (RGS) (1.0, 2.0, and 5.0 µM), respectively, for 24 and 48 hours (representative images). (**B**) Enzymatic LDH quantification in supernatants of HCC cells (PLC, Hep3B) treated with DMSO (0.0 µM RGS) as compared to 1.0, 2.0, 3.0, 4.0 and 5.0 µM RGS, respectively, for 24 and 48 hours. (**C**, **D**) Representative images of primary human hepatocytes (PHH) (C) and enzymatic LDH quantification in supernatants of PHH treated with DMSO as compared to 0.5, 1.0, and 5.0 µM RGS, respectively, for 48 hours (D). (**E**) Structural formula of rigosertib ("ON-01910.Na"). Data are represented as means ± SEM. OD: optic density. Ns: non-significant vs control. ^*^p < 0.05 vs control.

Initially, we investigated the effects of the benzyl styryl sulfone rigosertib (RGS, ON-01910) on viability of human HCC cell lines (PLC, Hep3B) *in vitro*. HCC cells revealed no signs of toxicity after treatment with 1–2 µM RGS for 48 hours. However, at higher doses (5 µM) microscopical analysis revealed morphologic changes in both HCC cell lines (Figure 1A). Lactate dehydrogenase (LDH) levels were slightly but significantly elevated in the supernatant of PLC cells treated with RGS doses of 3 µM or higher (Figure 1B). However, LDH levels were not significantly altered in Hep3B cells treated with up to 5 µM RGS (Figure 1B). In contrast to liver cancer cells, primary human hepatocytes (PHH) treated with RGS showed no signs of cytotoxic effects or changes in cell morphology and cell-cell contacts (Figure 1C). Moreover, RGS (doses up to 10 µm) treated PHH did not reveal alterations in lactate dehydrogenase (LDH) amounts as analyzed in cell supernatants (Figure 1D). These findings are in concordance with the known low toxicity profile of RGS in humans and on non-cancer cells [22–24]. In contrast, RGS treatment reveals toxic effects on HCC cells in moderate and high doses (>5 µM) but not in low doses (<5 µM).

### Effect of rigosertib on proliferation and RAS downstream signaling in HCC cells

**Figure 2:**
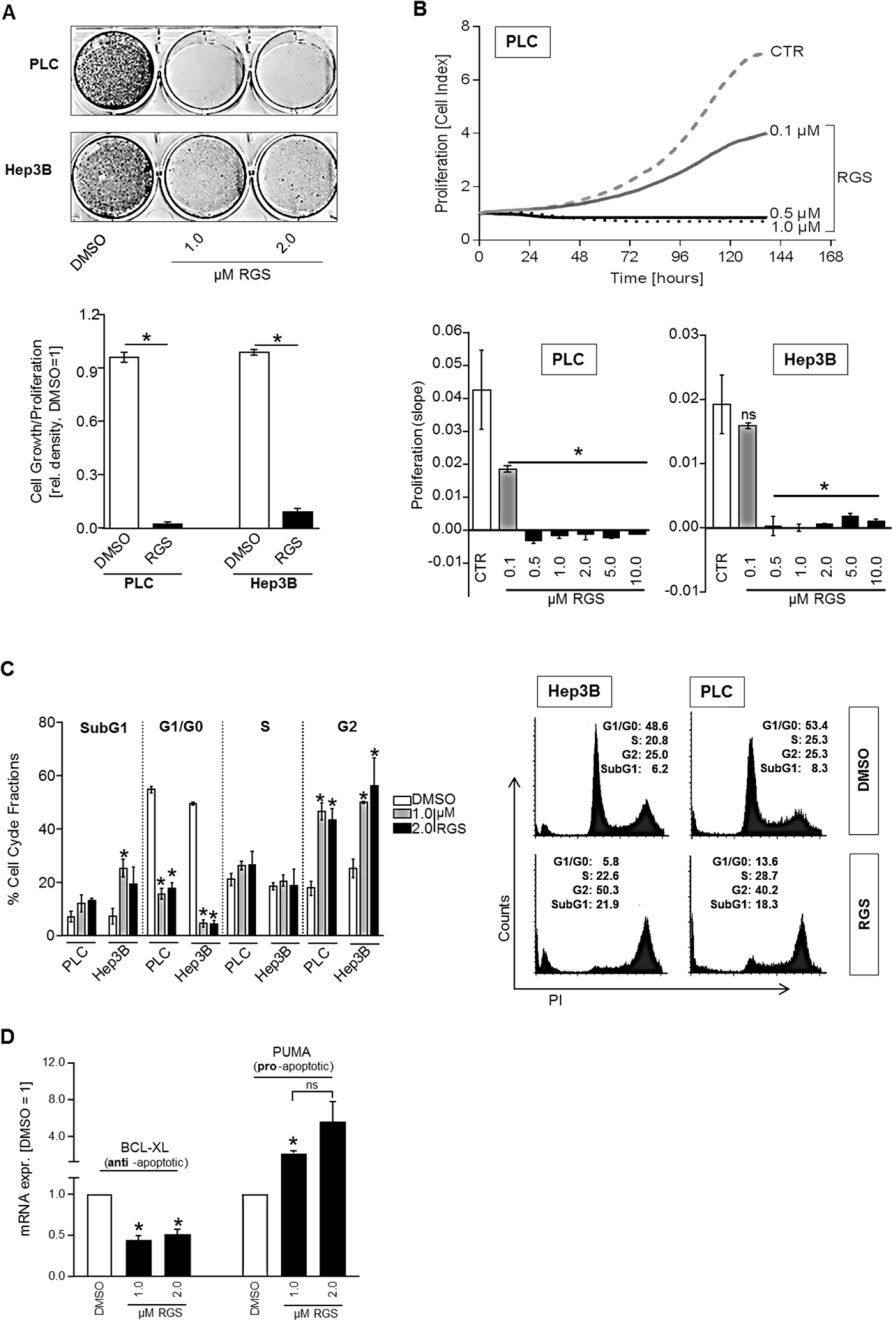
Effect of rigosertib on proliferation and cell cycle in HCC cells For functional analysis, HCC cells (PLC, Hep3B) were treated with DMSO (control=CTR) or different doses (0.1, 0.5, 1.0, 2.0, 5.0, 10.0 µM) of rigosertib (RGS), respectively. (**A**) Representative images (top panel) and densitometric quantification (bottom panel) of cultured HCC cells (PLC, Hep3B) (100,000 seeded cells in 6-well plates) that were treated as indicated for 6 days. (**B**) Real-time cell proliferation. Representative proliferation curves for PLC (top) and the summarized “slopes” of the curves depicting the increasing cell index (bottom) for PLC and Hep3B cells. (**C**) Fluorescence-activated cell sorting (FACS) analysis (propidium iodide staining (PI)). Prior to FACS analysis, cells were treated for 24 hours. Indicated is the percentage of cells in different cell cycle fractions (SubG1, G0/G1, S, and G2) (left panel), and representative images (1.0 µM RGS vs DMSO) (right panel). (**D**) BCL-XL (left side) and PUMA (right side) mRNA expression (qRT-PCR analysis) in HCC cells (PLC and Hep3B, the graph summarizes two pairs for each cell line) treated with 1-2 µM RGS or DMSO (control), respectively, for 24 hours. Data are represented as means ± SEM. OD: optic density. Ns: non-significant (vs DMSO). ^*^p < 0.05 (vs DMSO).

Functional analysis were performed using low concentrations of RGS (1–2 µM) to avoid toxicity-associated effects. RGS markedly reduced growth of HCC cells (Figure 2A). Also real-time cell proliferation assays showed that RGS strongly and dose-dependently reduced proliferation of both PLC and Hep3B HCC cell lines (Figure 2B). Indeed, significant inhibition of proliferation as compared to controls was already observed with doses as low as 0.1 µM RGS in PLC cells, and 0.5 µM RGS was sufficient to completely block cell prolifaration in both HCC cell lines (Figure 2B). Using fluorescence-activated cell sorting (FACS) analysis of cell cycle fractions, we found that RGS was sufficient to induce a G2/M cell cycle arrest in both PLC and Hep3B HCC cell lines (Figure 2C). Moreover, increased SubG1 cell cycle fractions indicated that RGS can also induce apoptosis in HCC cells (Figure 2C). RGS-mediated apoptosis induction therefore might explain the elevated LDH levels in cell supernatants as detected in PLC cells (Figure 1B). Accordingly, qRT-PCR analysis showed significant downregulation of the anti-apoptotic BCL-2-family member BCL-2-like-1 (BCL-XL) and significant upregulation of the pro-apoptotic BCL-2-family member p53-upregulated-modulator-of-apoptosis (PUMA), respectively, after rigosertib treatment (Figure 2D). Both BCL-XL and PUMA were shown to be strongly involved in HCC progression [25, 26].

**Figure 3:**
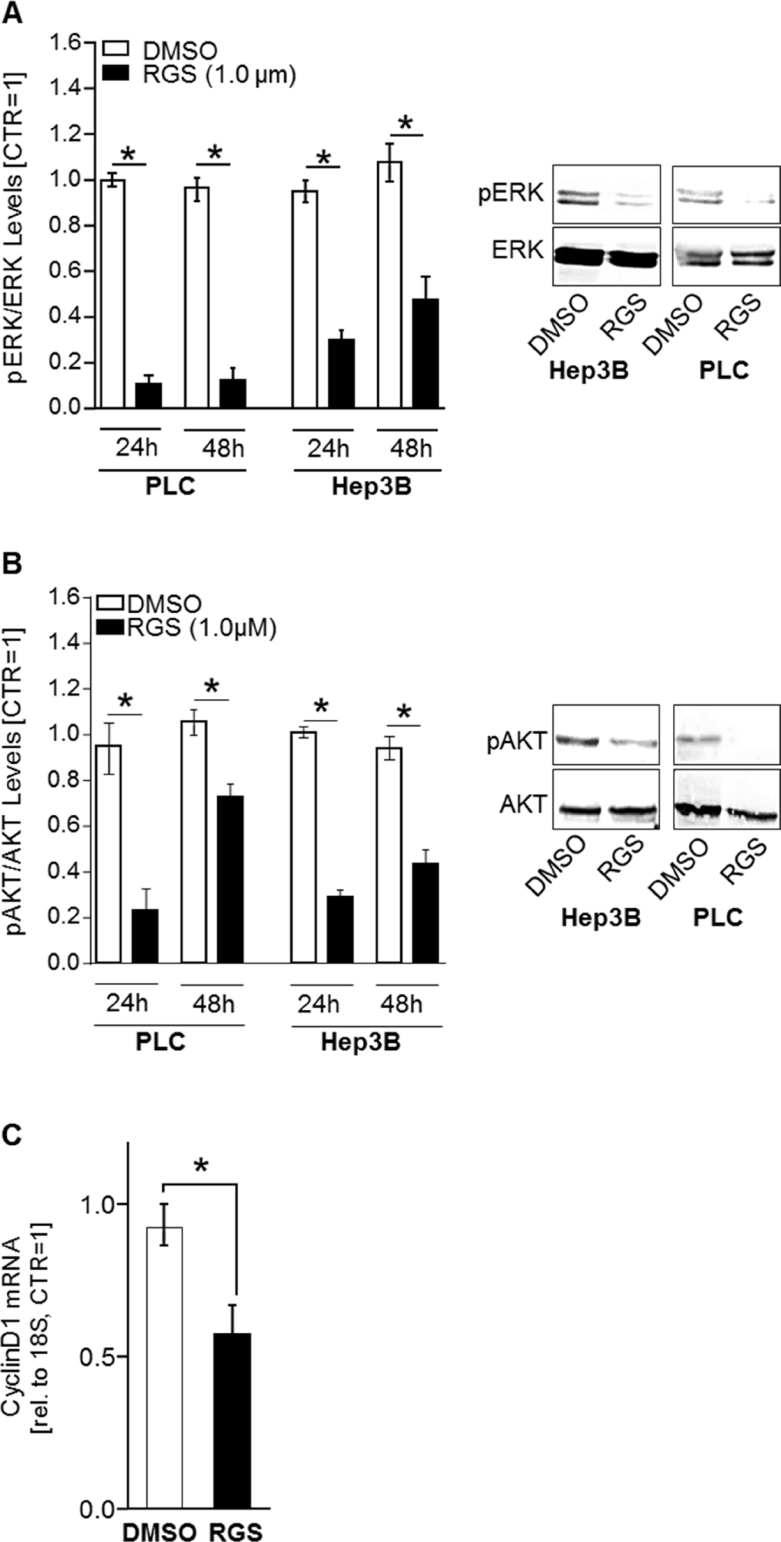
Rigosertib induced effects on RAS downstream signaling in HCC cells To reveal effects of rigosertib (RGS) on RAS downstream signaling pathways (PI3K-AKT and RAF-MEK-ERK), HCC cells (PLC and Hep3B) were treated with 1.0 µM RGS or DMSO, respectively, for 24 and 48 hours. (**A**) ERK-activation (phospho-ERK/ERK-relation) as quantified by densitometric western blot analysis (left panel) and according representative western blot images (right panel). (**B**) AKT-activation (phospho-aKT/AKT-relation) as quantified by densitometric western blot analysis (left panel) and according representative western blot images (right panel). pERK: phospho-ERK. pAKT: phospho-AKT. (**C**) CyclinD1 mRNA expression levels as quantified by qRT-PCR analysis in HCC cells (PLC and Hep3B, the graph summarizes two pairs for each cell line) treated with 1.0 µM RGS or DMSO (control), respectively, for 24 hours. ^*^ *p* < 0.05. Data are represented as means ± SEM.

RGS has been described to inhibit PLK1-activity, thereby inducing G2/M arrest in leukemia cells [27], but the exact mechanism of action was elusive. Recently, it has been discovered that RGS can interfere with RAS-signaling by binding to the RAS binding domains (RBD) of RAS-effector proteins (such as the rat fibrosarcoma (RAF) isoforms (ARAF, BRAF, and CRAF) and phosphatidylinositol 3-kinase (PI3K)) [18]. Apart from RAF-ERK-signaling, the PI3K-AKT-pathway is another RAS-regulated signaling axis that majorly contributes to HCC progression [28]. Since RAS proteins are common upstream regulators of both RAF-ERK and PI3K-AKT pathways, inhibition of RAS-signaling by RGS would be a desirable therapeutic approach for HCC treatment [2, 29, 30]. Western blot analysis showed that RGS significantly inhibited both ERK- (Figure 3A) and AKT-activation (Figure 3B) in HCC cells. Additionally, qRT-PCR analysis revealed that rigosertib treatment (1 µM, 24h) reduced CyclinD1 mRNA expression levels in HCC cells (Figure 3C). CyclinD1 is a known downstream target of RAS-ERK- and RAS-AKT-signaling pathways [31]. These data indicate that RGS acts as a novel and sufficient inhibitor of both PLK1 *and* RAS in HCC.

### Expression of PLK1 and HRAS in human HCC samples and cell lines

Athuluri-Divakar *et al.* recently found that the PLK1-inhibtor RGS actually acts as a RAS-mimetic that is able to reduce the transforming powers of mutant RAS isoforms including HRAS [18]. As mentioned above, next to PLK1, HRAS was described as a potential emerging oncogenic target in HCC [20, 21]. Therefore, we focused on the HRAS isoform to examine (combined) expression levels of both RGS targets (PLK1 and HRAS) in HCC.

**Figure 4:**
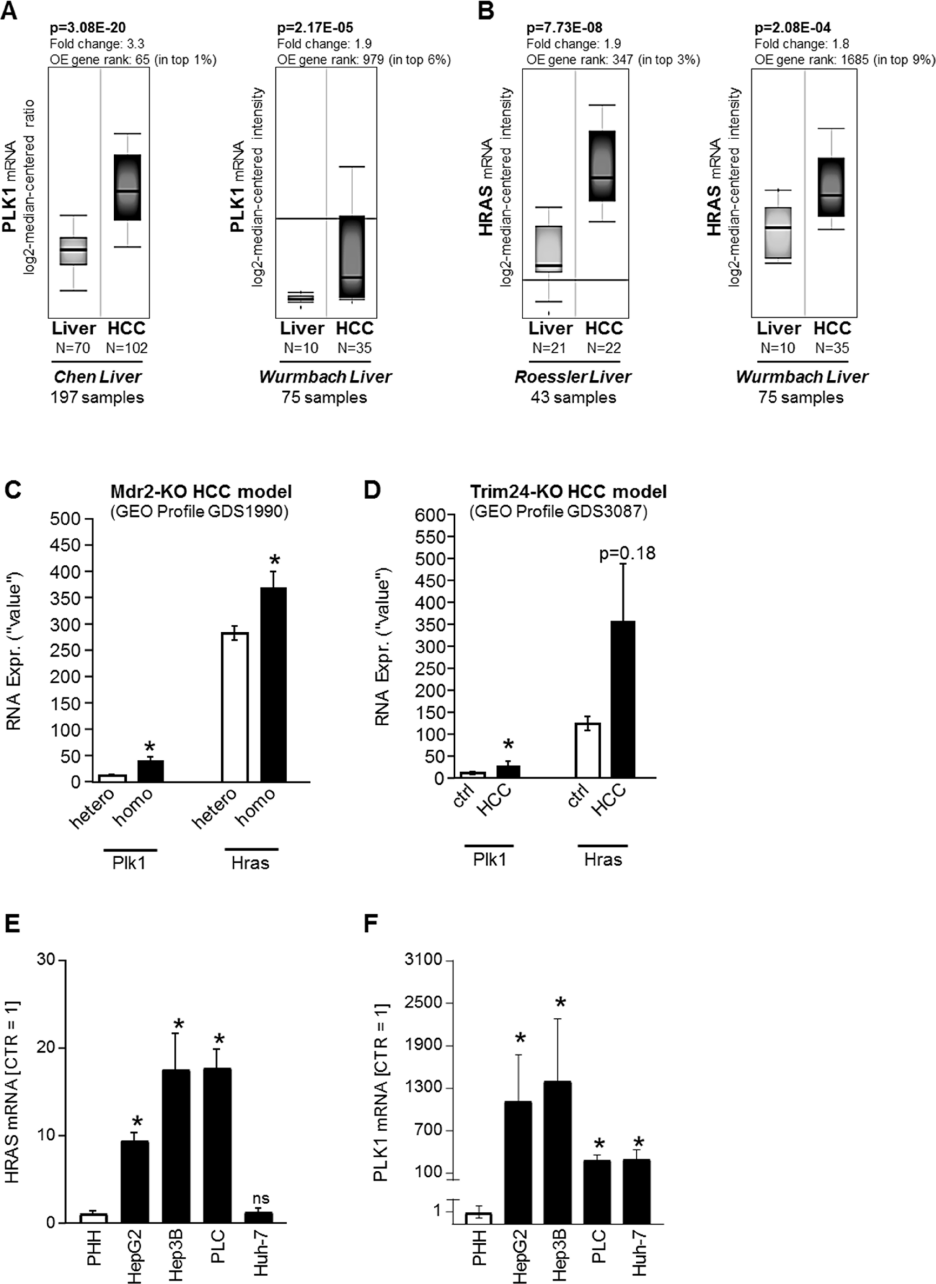
PLK1 and HRAS expression in HCC *in vivo* (**A**–**C**) PLK1 and HRAS (**B**) mRNA levels in liver tissues ("Liver") as compared to Hepatocellular carcinoma (HCC) patient tissues. Data were obtained from the OncomineTM cancer microarray database using the datasets “Chen Liver” (*N* = 197), “Wurmbach Liver” (*N* = 75) and “Roessler Liver” (*N* = 43). OE: Overexpression. (**C**, **D**) In silico analysis of RNA expression levels for PLK1 and HRAS was performed using GEO datasets (GEO profiles). (**C**) *In vivo* RNA expression levels (Plk1, Hras) in pre-cancerous stages in the murine Mdr2 knockout HCC model in both heterozygous (hetero, *N* = 6) and homozygous (homo, *N* = 6) knockouts (^*^p < 0.05 vs hetero). (**D**) *In vivo* RNA expression levels (Plk1, Nras, Hras, Kras) in livers from wild-type (ctrl, *N* = 5) as compared to HCC tumors (HCC, *N* = 5) derived from the Trim24-deficient spontaneous murine HCC model (ns: non-significant vs ctrl; ^*^p < 0.05 vs ctrl). (**E, F**) Quantitative RT-PCR analysis of HRAS (**E**) and PLK1 (**F**) mRNA expression levels in human HCC cell lines (HepG2, Hep3B, PLC, Huh-7) as compared to primary human hepatocytes (PHH) (^*^p < 0.05 vs PHH; ns: non-significant vs PHH).

Expression levels of PLK1 and HRAS in HCC patients were analyzed using the Oncomine™ human cancer microarray database [32]. Both PLK1 (Figure 4A) and HRAS (Figure 4B) were found to be strongly upregulated in HCC as compared to non-HCC liver tissues in several patient datasets (*Chen Liver*, 197 patient samples [33]; *Wurmbach Liver*, 75 patient samples [34]; *Roessler Liver*, 43 patient samples [35]). To gain insights into gene expression of PLK1 and HRAS-isoform in HCC, several GEO/GSE datasets were analyzed. To address gene expression levels during HCC development, a precancerous dataset comparing heterozygous and homozygous Mdr2 knockout (KO) mice was used. It has been shown before that the Mdr2-KO mouse is a valid model for human HCC development [36]. In this dataset, RNA expression levels of PLK1 and HRAS were significantly elevated in homozygous as compared to heterozygous knockout mice (Figure 4C). Moreover, PLK1 and HRAS were analyzed in a GEO dataset containing data of Trim24-deficient HCC samples and non-tumorous control liver tissues. Similar as Mdr2-KO mice, Trim24-deficient mice spontaneously develop HCC [37]. Also in this model, PLK1 and HRAS expression levels were elevated in HCC samples as compared to non-tumorous wild-type liver tissue (Figure 4D). Regarding the strong effects of rigosertib on HCC cell lines *in vitro* (Figures 1–3), qRT-PCR analysis of HRAS and PLK1 expression levels was also performed in HCC cell lines (HepG2, Hep3B, PLC, Huh-7) as compared to primary human hepatocytes (PHH), and revealed marked overexpression of both HRAS (Figure 4E) and PLK1 (Figure 4F) in HCC cells.

In summary, these data indicated that both PLK1 and HRAS expression levels increase during HCC development and remain elevated in (advanced) liver cancer *in vitro* and *in vivo*.

### Effects of PLK1 and HRAS expression levels on survival of HCC patients

**Figure 5:**
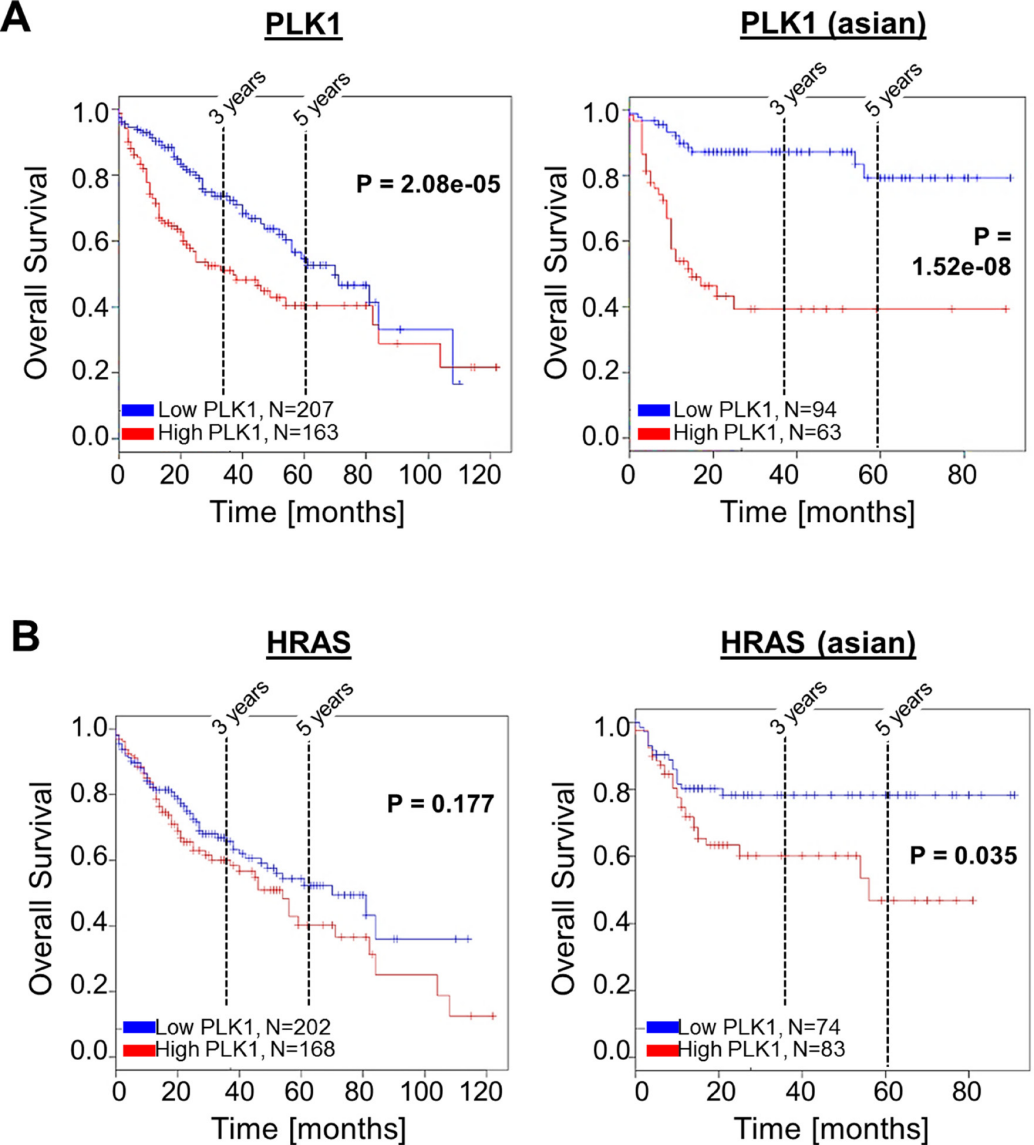
Effects of PLK1 and HRAS expression on HCC patient survival Representative Kaplan-Meier survival curve analysis was performed using the SynTarget / BioProfiling database for a TCGA HCC (LIHC) dataset (377 patient samples in total). (**A**) Kaplan-Meier curves for high vs low PLK1 expression in the complete patient cohort (left panel) and in a defined patient subset ("asian", right panel). (**B**) Kaplan-Meier curves for high vs low HRAS- expression in the complete patient cohort (left panel) and in a defined patient subset ("asian", right panel).

Next, we asked whether PLK1 and HRAS affect survival of HCC patients. Kaplan-Meier (overall) survival curves were analyzed using the *SynTarget/BioProfiling* database for a TCGA HCC (LIHC) dataset (377 patient samples) [38, 39]. We revealed that high PLK1 expression is a strong negative predictor for poor patient outcome. Significant differences were observed in the total patient cohort (*n* = 370, *p* < 0.0001) (Figure 5A, left panel). Interestingly, within the Asian population, even stronger effects of PLK1 overexpression on overall survival were detected (*n* = 157, *p* < 0.0001) (Figure 5A, right panel). In contrast, the expression level of HRAS was slightly but not significantly associated with patient overall survival in the total patient cohort (Figure 5B, left panel). However, within the Asian population, high HRAS expression significantly (*p* < 0.05) also correlated with low patient overall survival (Figure 5B, right panel).

**Figure 6:**
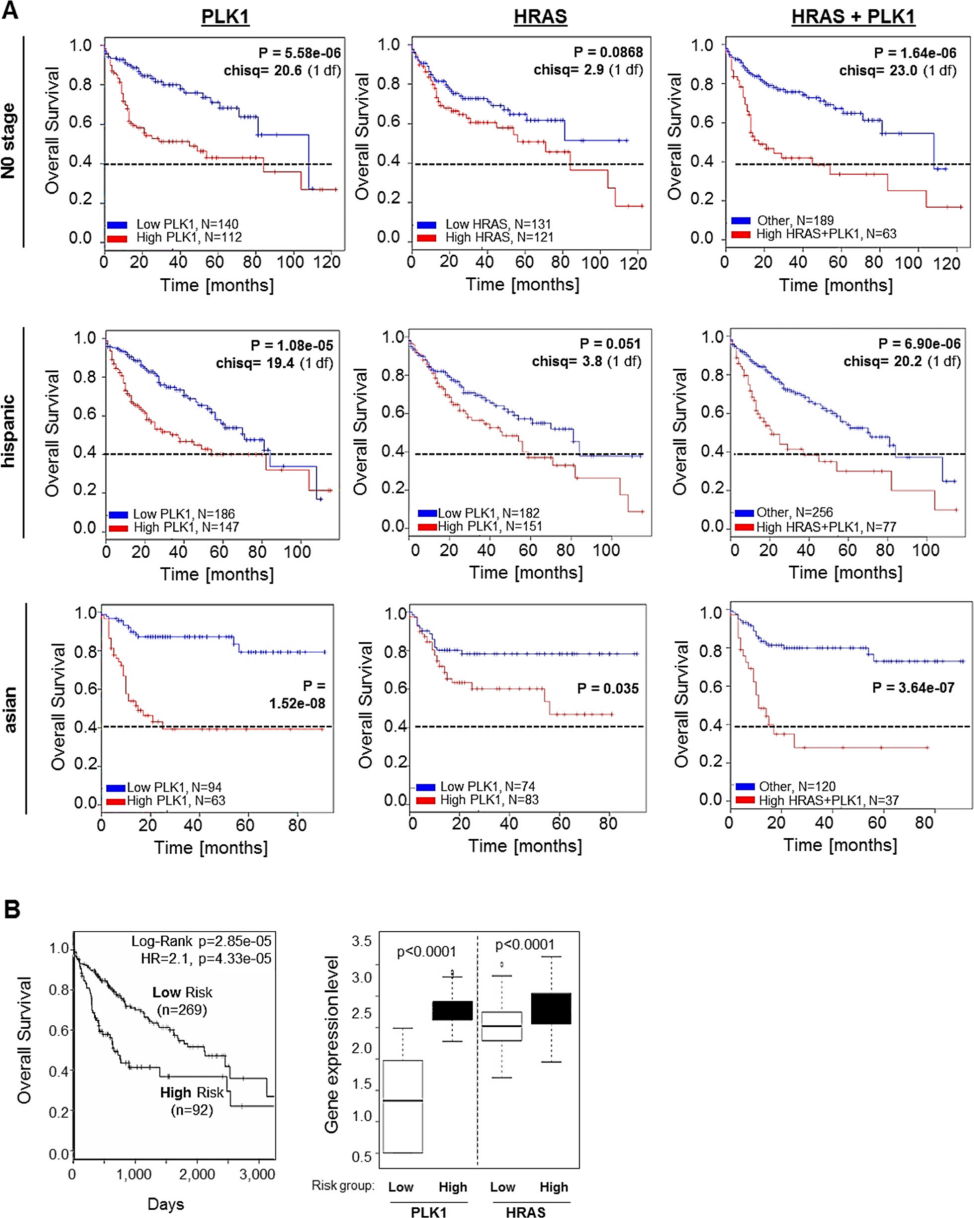
Combined effects of PLK1 and HRAS expression on overall survival of HCC patients (**A**) Representative Kaplan-Meier survival curve analysis was performed using the SynTarget / BioProfiling database for a TCGA HCC (LIHC) dataset (377 patient samples in total). The Kaplan-Meier curves were created for high vs low PLK1 expression (left panels), high vs low HRAS expression (central panels) and combined high PLK1 and HRAS expression (right panels) in different patient sub-cohorts ("N0-stage", Hispanic population, Asian population). (**B**) SurvExpress-Biomarker validation for cancer gene expression database analysis depicting a Kaplan-Meier curve for overall survival (left panel) according to stratification into “low risk” and “high risk” patient groups based on prognostic index. The right panel shows quantification of PLK1 and HRAS expression levels according to “low risk” and “high risk” patient groups.

Both PLK1 and RAS are targets of the novel and effective small molecule inhibitor rigosertib (Figures 1–3), and we found that PLK1 and HRAS are commonly upregulated in HCC (Figure 4) and independently affect patient overall survival (Figure 5) with interesting differences among different ethnic background. Therefore, potential combined effects of PLK1 and HRAS expression on HCC patient survival was analyzed in different patient subgroups. We found that combined high expression of PLK1 and HRAS had a stronger negative effect on patient overall survival as compared to high PLK1 or HRAS expression alone within the “N0 stage” subgroup and the Hispanic population (Figure 6A). Within the Asian subgroup, PLK1 and HRAS alone had very strong effects on patient survival (Figure 5). However, combined high expression of PLK1 and HRAS also tended to even lower overall survival (Figure 6A). In the total (mixed) patient cohort (with the majority of patients coming from the Asian subgroup), combined high PLK1 and HRAS expression showed a clear but not significant tendency to lower overall survival (data not shown). In line with these combined effects, TCGA (“The Cancer Genome Atlas”) data analysis using the “SurvExpress”-Biomarker validation for cancer gene expression database [40] revealed that stratification into low risk (*n* = 269) and high risk (*n* = 92) patient groups (based on prognostic index) shows reduced overall survival in the high risk group and marked overexpression of both PLK1 and HRAS (Figure 6B). Together, PLK1 and HRAS (both targets of rigosertib) additively/synergistically affect survival in HCC patients. Moreover, we observed interesting differences of these combined effects among several patient sub-cohorts.

## DISCUSSION

RAS is one of the most desirable molecular targets in cancer therapy, however, it was considered to be “undruggable” for a long time [4]. Novel techniques and approaches have renewed the efforts to combat RAS-signaling successfully, thereby promoting the “RAS-renaissance” [4, 41–44]. However, RAS-inhibitors are still poorly investigated in HCC, mostly because RAS proteins are uncommonly mutated in this cancer type [2, 45]. The RAS isoforms NRAS and KRAS are mutated in less than 2–4% of HCC and were not yet described as oncogenic targets in HCC [19]. In contrast, transcriptional and epigenetic activation mechanisms of (H)RAS proteins have been reported in experimental HCC models and human HCC tissues [20, 29, 30]. Moreover, activating HRAS mutations have been described in a murine model of non-alcoholic fatty liver disease [21], i.e. a condition which is increasingly recognized as a major risk factor for HCC development and progression. In our study, HRAS mutations were not found in PLC and Hep3B HCC cell lines, giving evidence that the emerging role of wild-type RAS proteins in cancer [46] is also relevant in HCC. Indeed, we found strong upregulation of HRAS expression levels in HCC cell lines as compared to hepatocytes and in HCC tissue samples as compared to non-tumorous liver tissues, pointing to transcriptional activation mechanisms rather than oncogenic mutations of HRAS in HCC.

Since RAS proteins are common upstream mediators of both RAF-ERK and PI3K-AKT-pathways, combinatory approaches targeting RAF/ERK- and PI3K/AKT-signaling could lead to major improvements in the management of HCC [28]. Recently, the novel benzyl styryl sulfone rigosertib (RGS) was shown to disrupt the binding of RAS-GTP with RAS binding domain (RBD) containing RAS-effectors [18]. Thereby, RGS reduced HRAS-induced malignant cell transformation [18]. In the present study we newly demonstrate that in HCC, RGS markedly reduced ERK- and AKT-activation, i.e. the two major effectors downstream of RAS-signaling. Moreover, we showed that RGS exerted strong inhibitory effects on proliferation in HCC cells. Most interestingly, RGS induced a G2/M cell cycle arrest unlike most RAF-inhibitors which cause a G1 cell cycle arrest [47]. The recent study of Athuluri-Divakar *et al.* demonstrated that RGS can mediate G2/M cell cycle arrest by disrupting RAS-mediated CRAF-phosphorylation at Serine 338, thereby inhibiting CRAF^Ser338^-mediated PLK1-activation resulting in a G2/M arrest [18]. PLK1 is involved in promoting cell cycle progression, and it is increasingly recognized as a crucial therapeutic target in HCC [11–17]. Novel, rapidly emerging findings (published 2016 and 2017) strongly support our results that PLK1 is indeed a promising therapeutic target gene for HCC [48, 49].

Here, we showed increased PLK1 expression during HCC development and in established HCC and revealed that PLK1 expression levels correlated with poor patient survival. Interestingly, PLK1 (and also HRAS) effects on survival were even more pronounced in the subgroup of Asian HCC patients. This might reflect differences in the etiologiy of underlining liver disease. Thus, viral hepatitis is more common in Asian population as compared to alcoholic/non-alcoholic fatty liver (which can be found in the majority of the caucasian population) [1, 2]. Future studies are needed to investigate in more detail whether PLK1 expression and function varies in HCC patients with different ethnic background, and if these differences might affect therapeutic responses and patient outcome. Such differences have been demonstrated for other oncogenic targets for HCC therapy such as immune checkpoint inhibition [50, 51]. Most interestingly, a very recent study reveals that mitotic checkpoint-associated genes including PLK1 are crucial key drivers that distinguish molecular subtypes among Asian HCC patients [52].

In addition to PLK1, our study revealed increased HRAS expression levels in HCC, and importantly, increased expression of both PLK1 and HRAS showed combined effects on HCC patient overall survival in different patient subgroups. This highlights the importance of these genes and associated pathways in HCC.

**Figure 7:**
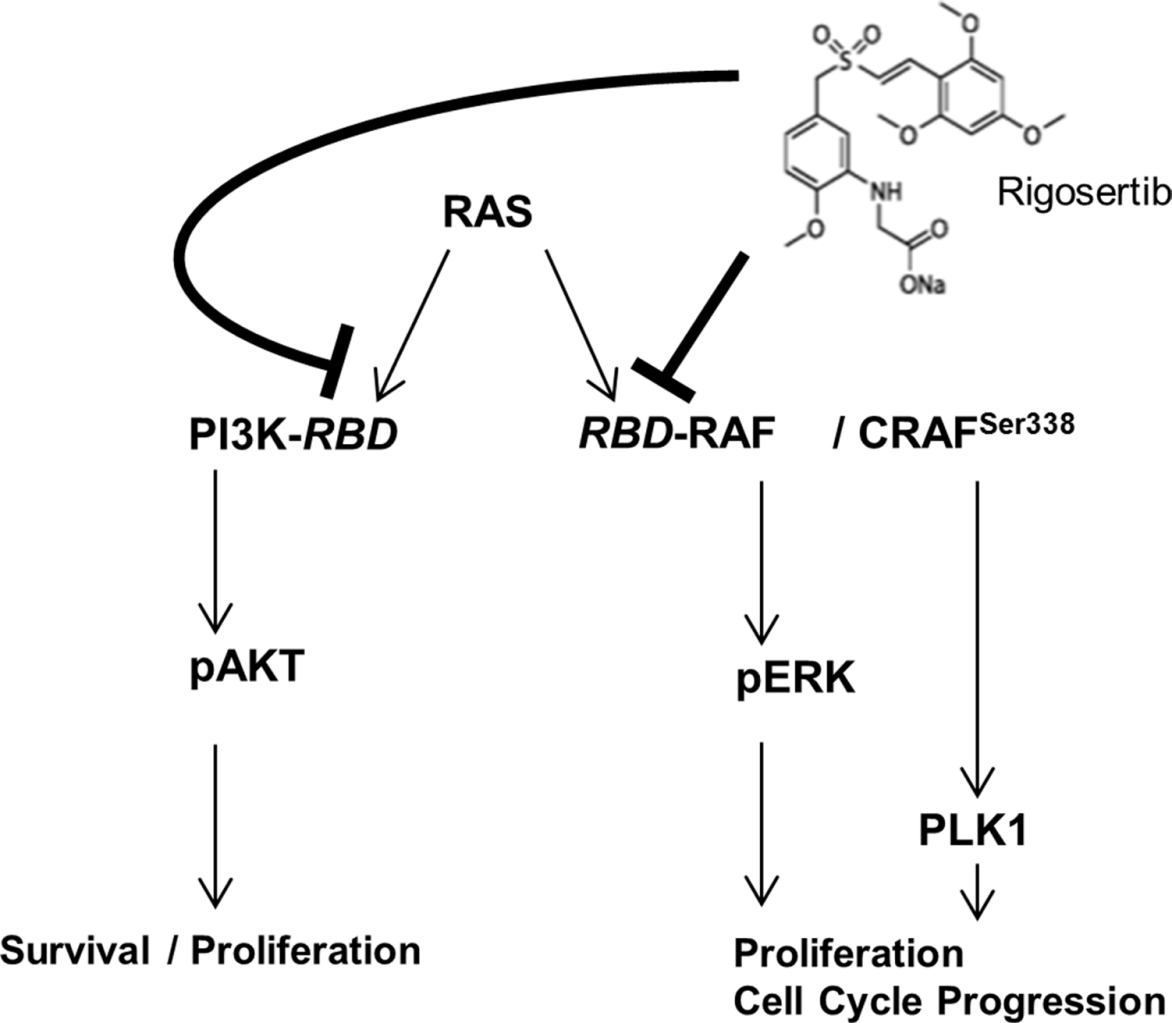
RGS induces G2/M cell cycle arrest and inhibits RAS-mediated ERK- and AKT-activation in HCC RGS most likely acts by inhibition of the RAS-downstream-pathways RAS-RAF-ERK and PI3K-AKT (by interfering with RAS-binding domains (RBD)). Moreover, CRAF-dependent PLK1-activation resulting in G2/M cell cycle arrest is affected, as shown in recent studies. Our data underline the importance of PLK1-activation as well as RAS-RAF-ERK and RAS-PI3K-AKT-signaling in HCC and suggest potential synergistic/additive effects that are commonly counteracted by RGS.

Our findings and studies by other groups indicate that RGS acts as a RAS-mimetic that inhibits two of the major RAS-signaling pathways in HCC, i.e. MAPK- and PI3K-signaling, herewith exhibiting strong anti-tumorigenic effects (Figure 7). Moreover, we found marked induction of G2/M cell cycle arrest followed by RGS treatment. This can be explained by the described inhibition of CRAF-mediated PLK1-activation [18, 53, 54] (Figure 7). This reveals a novel “dual-hit” therapeutic strategy for HCC, which could have prior effects as compared to single gene inhibition (i.e. using selective PLK1 inhibitors such as “volasertib”, which was recently proven to suppress HCC both *in vivo* and *in vitro* [49]). Moreover, RGS may counteract potential escape/resistance pathways (e.g. IGF-1R-mediated activation of RAS-signaling) that are activated in the presence of G1-arrest inducers such as sorafenib [55]. Importantly, RGS has already been tested in clinical phase II/III trials for myelodysplastic syndromes (MDS) and pancreatic cancer and showed low toxicity [47, 56, 57]. A recent study revealed that in high risk, late stage patients with MDS, the most common grade 3 or higher adverse events were anemia (18% of patients in the rigosertib group vs. 8% of patients in the control (i.e. best supportive care) group), thrombocytopenia (19% vs. 7%), and neutropenia (17% vs. seven 8%). Only three deaths (out of 184 patients in the rigosertib group) were attributed to rigosertib treatment [58]. Another recent phase II/III randomized study compared the efficacy and safety of rigosertib (RGS) plus gemcitabine (GEM) versus gemcitabine alone in patients with previously untreated metastatic pancreatic cancer and also revealed a low toxicity profile: Here, common grade 3 or higher adverse events were neutropenia (8% in the RGS plus GEM group versus 6% in the GEM group), hyponatremia (17% versus 4%), and anemia (8% versus 4%) [57]. In line with this, we found that effective anti-tumorigenic RGS doses did not exhibit toxic effects in primary human hepatocytes.

A recent randomized, double-blind, placebo-controlled phase 3 trial revealed that beyond the first line systemic therapy for advanced HCC (i.e. sorafenib) the novel multi-kinase inhibitor regorafenib can be used as an efficient second line therapeutic option for HCC patients who progressed on sorafenib treatment [10]. However, the survival benefit of regorafenib was only modest (2.8 months), and the authors of the study concluded that future trials should explore combinations of regorafenib with other systemic agents and third line treatments for patients who fail or who do not tolerate the sequence of sorafenib and regorafenib [10]. Our study suggests that rigosertib might be evaluated as such a potential systemic agent or as a third line therapeutic option, respectively, alone or in combination with sorafenib or regorafenib. In conclusion, the here presented “dual-hit” approach for HCC treatment has the potential to be quickly translated from bench to bed-side.

## MATERIALS AND METHODS

### Cells

The human HCC cell lines PLC (ATCC CRL-8024), Hep3B (ATCC HB-8064), HepG2 (ATCC HB-8065), and Huh-7 (ATCC PTA-4583) were cultured and used for expression and functional analysis as described [59]. The HRAS mutational status of the cell lines PLC and Hep3B (which were used for functional analysis) was “wild-type”. Primary human hepatocytes (PHH) were isolated and cultured as described in former studies [60].

For inhibition of RAS/PLK1 signaling, a recently developed non-ATP competitive small molecule inhibitor (ON-01910.Na, also “rigosertib”, referred to as “RGS”) (Selleck Chemicals, Houston, USA) was used (Figure 1E). Experiments were performed in HCC cells and PHH in different doses and for different time intervals as indicated. Controls were treated with according doses of solvent (DMSO).

### Protein analysis

Protein extraction and Western blotting was performed as described [47]. The following antibodies were used: anti-phospho-ERK (1 in 4,000 dilution; Cell Signaling, Frankfurt am Main, Germany), anti-ERK (1 in 1,000 dilution; Cell Signaling), anti-phospho-AKT (1 in 2,000 dilution; Cell Signaling) and anti-AKT (1 in 2,000 dilution; Cell Signaling). Immunoreactions were visualized using NBT/BCIP (Sigma-Aldrich) staining. Computer based densitometry of the scanned Western blot images was performed for quantification (“ImageJ” (National Institutes of Health, USA)).

### Quantitative RT-PCR analysis

Quantitative RT–PCR was performed as described [46]. Annealing and melting temperatures were optimized for each primer set. Real-time quantitative PCR (qRT-PCR) was performed using the following primer pairs: 18S rRNA (5′-GCA ATT ATT CCC CAT GAA CG-3′ and 5′-GGG ACT TAA TCA ACG CAA GC-3′), BCL-XL (BCL2L1) (5′-ATC CAC TCT ACC CTC CCA CC-3′ and 5′- AGG GAG GCT AAG GGG TAA GG-3′), CyclinD1 (5′-GCC TGT GAT GCT GGG CAC TTC ATC TG-3′ and 5′-TTT GGT TCG GCA GCT TGC TAG GTG AC-3′), HRAS (5′-TGG TGG GGA ACA AGT GTG AC-3′ and 5′-TTG TGC TGC GTC AGG AGA G-3′), PLK1 (5′- TGA CTC AAC ACG CCT CAT CC-3′ and 5′-GCT CGC TCA TGT AAT TGC GG-3′) and PUMA (5′-ACC TCA ACG CAC AGT ACG AG-3′ and 5’-ATG GTG CAG AGA AAG TCC CC-3′). Sanger sequencing was performed using the following primer pair (covering all three HRAS-hotspots (G12, G13, Q61)): 5′-TAT AAG CTG GTG GTG GTG GG-3′ and 5′-AAC ACA CAC AGG AAG CCC TC-3′. Relative gene expression was normalized to mRNA levels using the comparative cycle threshold (Ct) method.

### Analysis of cell proliferation

The xCELLigence System (Roche) was used for analysis of real-time cell proliferation (using “E-Plates”) as described before [61]. Analysis of cell cycle fractions was performed using fluorescence activated cell sorting (FACS) as previously described [62].

### Lactate dehydrogenase assay

For quantification of lactate dehydrogenase amounts in cell supernatants, after 24 hours, lactate dehydrogenase (LDH) assays were performed *using* enzymatic techniques as described [63].

### *In silico* analysis

*In silico* analysis of RNA expression levels for PLK1 and HRAS was performed using GEO datasets (GEO profiles). A murine Mdr2 knockout HCC model in both heterozygous (hetero, *N* = 6) and homozygous (homo, *N* = 6) knockouts was used. Different genes were analyzed (GEO datasets: “GDS1990 / 1448191_at”: PLK1, “GDS1990 / 1422407_s_at”: HRAS) in precancerous stages. The Mdr2-KO mouse serves as a model for beta-catenin-negative subgroup of human HCCs characterized by down-regulation of multiple tumor-suppressor genes [36]. Moreover, the Trim24-KO murine HCC model was used to determine gene expressions in another GEO dataset (“GDS3087 / 1448191_at”: PLK1, “GDS3087 / 1424132_at”: HRAS) in wild-type as compared to Trim24 deficient mice. Trim24 knockout mice spontaneously develop HCCs [37]. Kaplan-Meier survival curve analysis for either one or two genes were analyzed using the *SynTarget/BioProfiling* database for a TCGA HCC (LIHC) dataset (377 patient samples in total) as described [38, 39]. Oncomine™ cancer microarray database analysis for gene expressions was performed using the website (https://www.oncomine.org/). The “SurvExpress-Biomarker validation for cancer gene expression” database (http://bioinformatica.mty.itesm.mx:8080/Biomatec/SurvivaX. jsp), was used for analysis of a hepatocellular carcinoma TCGA dataset as described [40].

### Statistical analysis

Results are expressed as mean ± SEM. The Student’s *t*-test or one-way ANOVA, if appropriate, were used for group comparisons. The level of significance was *p* < 0.05 (using abbreviations “ns”: not significant; “*”: *p* < 0.05). The number of independent experiments was n ≥2–4. Analysis was performed using the GraphPad Prism Software (GraphPad Software, Inc., San Diego, CA, USA).

## Abbreviations

AKT: v-akt murine thymoma viral oncogene
CTR: control
ERK: extracellular-signal regulated kinase
HRAS: Harvey rat sarcoma virus oncogene
KR, MAPK: mitogen-activated protein kinase
PI3K: phosphatidylinositol-4,5-bisphosphate 3-kinase
RGS: rigosertib
SF: sorafenib
vs: *versus*
